# Supplementary material for: EnzML: multi-label prediction of enzyme classes using InterPro signatures
Source: BMC Bioinformatics. 2012 Apr 25;13:61. doi: 10.1186/1471-2105-13-61 (PMC3483700; doi:10.1186/1471-2105-13-61)
Supplement: Addtional file 5 — The Java code to format the data files, evaluate and predict. The file enzml_java_code.tar.gz contains the Java code used to format database data to ARFF and XML formats, to execute cross and train-test (jackknife) evaluations and to record evaluation results to database. More information is included in the readme.txt file and the Javadoc files. The code can be used with a MySQL database. To use a different database software, other JDBC drivers might be required. [file 1471-2105-13-61-S5.gz › java_code/enzml2011/doc/uk/ac/ed/inf/taweka/mulan/learn/MulanCrossExperimenter.html]

MulanCrossExperimenter


---


|  |  |  |
| --- | --- | --- |
| **Overview** \| **Package** \| **Class** \| **Use** \| **Tree** \| **Deprecated** \| **Index** \| **Help** | |  |
| **PREV CLASS**   **NEXT CLASS** | **FRAMES**    **NO FRAMES**     **All Classes** |  |
| SUMMARY: NESTED \| FIELD \| CONSTR \| METHOD | DETAIL: FIELD \| CONSTR \| METHOD |  |


---


## uk.ac.ed.inf.taweka.mulan.learn Class MulanCrossExperimenter

```
java.lang.Object
  uk.ac.ed.inf.taweka.Initialised
      uk.ac.ed.inf.taweka.mulan.learn.MulanCrossExperimenter
```

**All Implemented Interfaces:**: Initialisable

**Direct Known Subclasses:**: TrainTestExperimenter

---

``` public class MulanCrossExperimenter extends Initialised ```

Using Mulan (multi-label machine learning), this class initialises and
executes either Train-Test evaluation (if two different arff records are
given) (calling `MulanTrainTestEvaluator`) or cross-evaluations (calling
`MulanCrossEvaluator`) if only one arff record is given. Initialisation
includes verifying input (arff and xml files) and checking output locations
(for saving results to file and database).

**Version:**
:   14 Jun 2010

**Author:**
:   Luna De Ferrari luna.deferrari-at-ed.ac.uk

---

**Field Summary**

**Fields inherited from class uk.ac.ed.inf.taweka.Initialised**

`m_initialisationIsCorrect`


**Constructor Summary**

`MulanCrossExperimenter(java.lang.String resultsDbPropsFile,
java.lang.String arffRecordDbProps,
int trainArffRecordId,
MulanLearner learner)`


| **Method Summary** | |
| --- | --- |
| `void` | `crossEvaluateAndSaveResults(int folds)`             Cross validates and saves results to database |
| `void` | `evaluateAndSave(int folds)` |
| `MulanCrossEvaluator` | `getEvaluator()` |
| `uk.ac.ed.inf.utils.database.DbManager` | `getExperimentDbManager()` |
| `MulanLearner` | `getLearner()`             public String getExperimentPropsPath() { return m\_experimentPropsFile; } |
| `ResultsSaver` | `getResultsSaver()` |
| `MulanArffRecord` | `getTrainArffRecord()` |
| `static void` | `main(java.lang.String[] args)` |

**Methods inherited from class uk.ac.ed.inf.taweka.Initialised**

`initialisationIsCorrect, setInitialisationIsCorrect`

**Methods inherited from class java.lang.Object**

`equals, getClass, hashCode, notify, notifyAll, toString, wait, wait, wait`

**Constructor Detail**

### MulanCrossExperimenter

```
public MulanCrossExperimenter(java.lang.String resultsDbPropsFile,
                              java.lang.String arffRecordDbProps,
                              int trainArffRecordId,
                              MulanLearner learner)
```


**Method Detail**

### crossEvaluateAndSaveResults

```
public void crossEvaluateAndSaveResults(int folds)
```

:   Cross validates and saves results to database

---


### evaluateAndSave

```
public void evaluateAndSave(int folds)
```

---


### getEvaluator

```
public MulanCrossEvaluator getEvaluator()
```

---


### getExperimentDbManager

```
public uk.ac.ed.inf.utils.database.DbManager getExperimentDbManager()
```

---


### getLearner

```
public MulanLearner getLearner()
```

:   public String getExperimentPropsPath() { return m\_experimentPropsFile; }

---


### getResultsSaver

```
public ResultsSaver getResultsSaver()
```

---


### getTrainArffRecord

```
public MulanArffRecord getTrainArffRecord()
```

---


### main

```
public static void main(java.lang.String[] args)
```


---


|  |  |  |
| --- | --- | --- |
| **Overview** \| **Package** \| **Class** \| **Use** \| **Tree** \| **Deprecated** \| **Index** \| **Help** | |  |
| **PREV CLASS**   **NEXT CLASS** | **FRAMES**    **NO FRAMES**     **All Classes** |  |
| SUMMARY: NESTED \| FIELD \| CONSTR \| METHOD | DETAIL: FIELD \| CONSTR \| METHOD |  |


---
